# The Evolution of Connectivity in Metabolic Networks

**DOI:** 10.1371/journal.pbio.0030228

**Published:** 2005-06-28

**Authors:** Thomas Pfeiffer, Orkun S Soyer, Sebastian Bonhoeffer

**Affiliations:** **1** Computational Laboratory, ETH Zurich, Zurich, Switzerland,; **2** Ecology and Evolution, ETH Zurich, Zurich, Switzerland; Princeton UniversityUnited States of America

## Abstract

Processes in living cells are the result of interactions between biochemical compounds in highly complex biochemical networks. It is a major challenge in biology to understand causes and consequences of the specific design of these networks. A characteristic design feature of metabolic networks is the presence of hub metabolites such as ATP or NADH that are involved in a high number of reactions. To study the emergence of hub metabolites, we implemented computer simulations of a widely accepted scenario for the evolution of metabolic networks. Our simulations indicate that metabolic networks with a large number of highly specialized enzymes may evolve from a few multifunctional enzymes. During this process, enzymes duplicate and specialize, leading to a loss of biochemical reactions and intermediary metabolites. Complex features of metabolic networks such as the presence of hubs may result from selection of growth rate if essential biochemical mechanisms are considered. Specifically, our simulations indicate that group transfer reactions are essential for the emergence of hubs.

## Abstract 2

Computer simulations show how the complex organization of metabolic networks can arise from selection for a simple trait such as growth rate.

## Introduction

Metabolic networks belong to the best-studied biochemical networks. They consist of metabolites and of biochemical reactions transforming these metabolites into each other. In contrast to other biochemical networks such as signal transduction networks and genetic networks, complete network topologies can be easily obtained from annotated genomes. It has recently been reported that the connectivity of metabolic networks follows approximately a power law, i.e., the frequency, *P*(*k*)*,* of metabolites participating in *k* reactions is given by *P*(*k*)*∼k^−γ^*, where *γ* is a constant coefficient [1–4]. This distribution implies that although most metabolites are involved in only few reactions, some metabolites serve as “hubs” and are involved in many reactions. These hubs are more frequent then would be expected, for example, in random networks. Interestingly, many hubs, such as ATP, NADH, glutamate, coenzyme A, and their derivates, serve as key compounds in the transfer of specific biochemical groups, such as phosphate groups, redox equivalents, amino groups, and acetyl groups.

Using computer simulations we here study whether hubs emerge in a widely accepted scenario for the evolution of metabolic networks. This scenario has originally been proposed by Kacser and Beeby [5] and considers an early stage in the evolution of life. It is based on the assumption that cells exist with (1) membranes that separate the interior of the cell from the environment, (2) a metabolism that allows cells to transform compounds taken up from the environment into those that are required for the formation of biomass, and (3) genes coding for enzymes that catalyze these biochemical reactions. Because of the low coding capacity and because it is unlikely that a large number of specialized enzymes emerge de novo, it is plausible to assume that these ancestral cells had only a few enzymes with broad specificities. The broad specificity allowed catalyzing all essential reactions at the cost of a low turnover for any single biochemical reaction. The ancestral cells are assumed to have been selected for growth rate and evolve by mutations affecting the kinetic properties of the enzymes and occasional gene duplications. This lead to the emergence of networks with highly specialized enzymes as observed in modern organism.

A number of alternative scenarios for the evolution of novel enzymes and metabolic pathways have been proposed [6]. One scenario for the evolution of metabolism in the early history of life has been proposed by Horowitz [7] and is referred to as “backwards evolution.” This scenario starts in an environment containing a large number of metabolites. Therefore only a low number of reactions are required to produce a useful product from one of these metabolites. As metabolites get depleted from the environment, longer pathways evolve in a reverse fashion, connecting the product to more distant metabolites present in the environment. A further scenario for the evolution of novel enzymes and metabolic pathways is referred to as “enzyme recruitment” [8]. This scenario is related to the scenario proposed by Kacser and Beeby. However, it does not apply to the initial emergence of metabolic networks early in the history of life, because it assumes that a core metabolic network with specialized enzymes is already present. Novel pathways emerge by recruiting enzymes from existing pathways. Analysis of distribution patterns of homologous enzymes in metabolic networks and of reactions catalyzed by different enzymes of the same family support enzyme recruitment rather than backwards evolution [6,9], but are also compatible with the Kacser and Beeby scenario. We therefore argue that this scenario represents a plausible mechanism for the evolution of metabolic networks in early organisms. It remains to be shown whether it indeed leads to the emergence of metabolic networks with connectivity distributions similar to those observed in nature. Based on the scenario of Kacser and Beeby, we therefore develop a model that allows simulating the evolution of metabolic networks and compare the resulting networks with natural networks.

### Assumptions

We simulate the evolution of metabolic networks based on the following assumptions:

Because of the importance of group transfer in metabolic networks we assume that metabolic networks are group transfer networks (Figure 1). Metabolites consist of biochemical groups. Enzymes catalyze the transfer of groups between metabolites. Additionally there are transporters that catalyze the uptake or excretion of metabolites. Some of the metabolites are involved in the formation of biomass. The concentrations of these metabolites determine the rate of biomass formation. Biomass formation results in growth. The growth rate is given by the rate and the costs of biomass formation (i.e., the cost for the synthesis of enzymes and other compounds in the cell, see Materials and Methods). The dynamics of the metabolites is described by a set of ordinary differential equations for the metabolite concentrations and is based on the kinetic properties of the reactions and transport processes. Additionally, growth leads to the dilution of metabolites at a rate equal to the growth rate. The system has therefore no conservation relations, and metabolites that are not involved in a reaction or transport process have a steady-state concentration of zero. There are two ways in which a metabolite can have a non-zero steady state. Either it participates in at least two reactions—one of which produces the metabolite, while the other one consumes the metabolite–or a metabolite is produced by a single reaction and is diluted due to growth. Fitness is assumed to be proportional to the growth rate at steady state of the system. Thus fitness is a dynamic property of the entire network.

**Figure 1:**
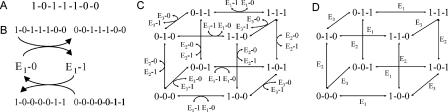
Structure of the Initial Network (A) Metabolites are assumed to consist of *N* different biochemical groups. Metabolites can be denoted as binary strings, because each group can be either present or absent. In the example given, the metabolite carries groups 1, 3, 4, and 5 whereas all other groups are absent. (B) Enzymes catalyze the transfer of specific biochemical groups between metabolites. For the group transfer we assume a ping-pong–like mechanism that consists of two half-reactions. In the first half-reaction, the group is transferred from a donor to the enzyme. Thereby the donor is transformed into its corresponding acceptor. In the second half-reaction, the enzyme transfers the group to another acceptor, thereby transforming it into its corresponding donor. We assume that a half-reaction follows linear reversible kinetics. This results in Michaelis-Menten–like kinetics for the transfer of a group from one metabolite to another. For the initial network we assume that enzymes are specific with regard to the group but unspecific with regard to donors and acceptors, i.e., they transfer a specific biochemical group from any donor to any acceptor. (C) The initial network consists of 2*^N^* metabolites. A single half-reaction transforms the metabolites into one of the *N* neighbours, such that the initial network resembles an *N*-dimensional hypercube. Note, however, that for the transfer of a group, two edges (i.e., half-reactions) in the cube have to be coupled. (D) To study the impact of group transfer on the connectivity distribution of the simulated networks, we performed simulations with monomolecular reaction networks, in which groups are added and removed, but not transferred, by the enzymes. The initial network has a similar structure. However, in contrast to the group transfer network, each reaction can be performed without coupling to another reaction.

We assume that initially all enzymes and all transporters are unspecific and allow small mutations in their kinetic properties. These mutations increase the rate constant of a single reaction while decreasing the rate constants of all other reactions of an enzyme or transporter. Alternatively they decrease the rate constant of a single reaction while increasing the rate constants of the other reactions. In addition to mutations affecting kinetic properties, we assume that there are gene duplications (or deletions) that increase (decrease) the dosage of enzymes and transporters. Changing the dosage of an enzyme has two opposing effects on fitness: An increased dosage increases the fluxes in the network, but it is also associated with increased metabolic costs of biomass formation. Duplications also enable enzymes to diverge and specialize on different reactions. Note that if a multifunctional enzyme has only a small impact on biomass formation, duplication can be detrimental provided that the metabolic costs of the additional copy are larger than the benefits in terms of increased growth rate. Consequently, enzymes and transporters do not necessarily specialize completely over the course of evolution. On the other hand, duplications can be beneficial if enzymes or transporters are already completely specialized. Therefore multiple identical copies of completely specialized enzymes and transporters can evolve provided they have a large impact on biomass formation.

In our simulations, we assume that there are seven different biochemical groups that characterize each metabolite. Because a metabolite can either carry one or no copies of each group, we have an initial network of 128 metabolites. We initiate the simulations with seven unspecific enzymes that catalyze the transfer of one of the biochemical groups from any donor to any acceptor, and with a single unspecific transporter that catalyzes the transport of all metabolites. Sixteen randomly chosen metabolites are involved in biomass formation. For the production of biomass, it is necessary to have at least one donor and acceptor of each group as external metabolites. We therefore assume that a minimal set of two metabolites, namely X0 and X127, is present in the environment. For reasons of computational tractability, we let the network evolve by fixation of the most beneficial mutations rather than random mutations. Duplications and deletions, because we assume that they are rare compared to mutations that alter the kinetic properties, are only considered if no further beneficial kinetic mutations are possible. A detailed description of the computer simulations is given in Materials and Methods.

## Results

A sample simulation is shown in Figure 2. The simulation indicates that the scenario proposed by Kacser and Beeby [5] indeed leads to the evolution of metabolic networks with specialized enzymes (Figure 2A). The emerging network consists of 23 different reactions and seven transport processes that are catalyzed by fully specialized enzymes and transporters (Figure 2C). The enzymes and transporters are present in multiple identical copies. The network contains only a fraction of the metabolites present in the initial network (33 of 128). The remaining metabolites become unconnected as the reactions specialize, i.e., they do not serve as substrate or product of reactions present in the emerging network. Figure 2B shows the connectivity distribution of the metabolites in the emerging network. The connectivity includes participation in enzymatic reactions, transport processes, and biomass formation. Most metabolites have a connectivity of two. In this simulation there are no metabolites that participate in only one reaction or transport process. Some of the metabolites, such as X0, X16, X18, X126, and X127, are involved in a comparably large number of reactions. These metabolites “monopolize” the transfer of specific biochemical groups. One of the groups, for example, is always transferred from metabolite X127 to a number of different acceptors. Hence, it seems to be beneficial that at least some of the metabolites specialize in the transfer of specific groups by acting as the only donor or acceptor.

**Figure 2:**
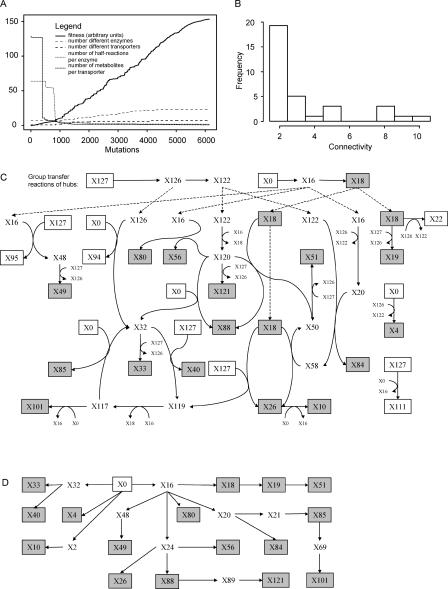
Sample Simulation for the Evolution of Metabolic Networks (A) The initial network consists of 128 metabolites, seven unspecific enzymes (each of which transfers one of the seven biochemical groups that metabolites carry), and a single unspecific transporter. Within the course of evolution, the enzymes and transporter duplicate and increase in specificity (i.e., the number of half-reactions per enzyme and of metabolites per transporter decreases). The emerging network consists of 23 enzymatic reactions and seven transport processes. In the sample simulation, all enzymes and transporters in the emerging network are highly specific, i.e., the enzymes catalyze only two half-reactions, and the transporters transport single metabolites. The emerging network contains only 33 metabolites. The remaining metabolites are not involved in the emerging network. (B) Connectivity distribution of the emerging group transfer network. Most metabolites are involved in only two reactions. However, a few metabolites are highly connected. (C) Pathway scheme of the emerging group transfer network. The metabolites X0 and X127 are taken up from the environment, whereas metabolites X4, X22, X94, X95, and X111 are excreted into the environment (white boxes). The network eventually transforms metabolites X0 and X127 into those metabolites that are involved in biomass formation (grey boxes). Interestingly, metabolite X4 is excreted although it is involved in biomass formation. Note that some half-reactions evolve, such as the one from X127 to X126, and monopolize the transfer of a specific group (in this case the first group in the binary string). These metabolites are involved in many reactions and therefore have high connectivity. The group transfer reactions of these hubs are summarized in the first line of the pathway scheme. The emerging group transfer network is much more complex than the corresponding monomolecular reaction network (see Figure 2D) and even includes a cycle (X32 → X119 → X117 → X32), with the net reaction of X0 + X16 + X127 → X18 + X40 + X85). (D) Pathway scheme of an emerging monomolecular reaction network. Although the network produces the same set of metabolites involved in biomass formation as the group transfer network shown in Figure 2C, it has a much simpler, tree-like structure. Additionally, the network has no obvious hubs.

To obtain more precise data for the connectivity distribution, we analysed 40 replicate simulations. The results of these simulations are similar to the sample simulation shown in Figure 2. Most of the enzymes and transporters of the emerging networks specialize completely, i.e., catalyze two half-reactions or the transport of a single metabolite. However, a small fraction of the enzymes in the emerging networks catalyze more than two half-reactions, i.e., are not fully specialized. These enzymes have only a weak impact on the rate of biomass formation and therefore cannot duplicate and specialize fully (see Assumptions). Most of the fully specialized enzymes and transporters are present in multiple identical copies. As in the sample simulation, the emerging networks contain only a small fraction of the metabolites present in the initial networks. Most of these metabolites are involved at least in two reactions or transport processes. Occasionally we observe metabolites that participate in only a single reaction (see Assumptions). Figure 3A shows the connectivity distribution over all emerging networks. Metabolites that are participating in fewer than two reactions are not included in the graph. The average maximum likelihood estimate [10] for the power-law coefficients of the individual networks is γ = 2.63 (±0.17). However, the overall connectivity distribution deviates strongly from a power law (Figure 3B). In particular, the overall distribution has more hubs than expected in the connectivity range between eight and 12, but has fewer hubs than expected with a connectivity of more than 12. This disagreement between the emerging networks and natural networks may arise because the networks emerging in our simulations are much smaller than the networks analyzed in the literature [2,3,11]. We therefore tested whether the simulated networks are consistent with subnetworks of similar size derived from real metabolic networks. These networks were obtained from the KEGG database [12]. The KEGG database displays several functional metabolic subnetworks such as glycolysis, the tricarboxylic acid cycle, or amino acid synthesis for a wide range of organisms. We analyzed 22 subnetworks from*Escherichia coli,* each containing between 24 and 40 metabolites. The overall connectivity of these networks is shown in Figure 3C. Note, that in order to compare the connectivity distributions of the simulated and the natural networks, we treat the natural networks as independent networks, i.e., a metabolite that is present in two different networks is treated as two different metabolites. As in the simulated networks, we consider only metabolites that are involved in more than one reaction. A comparison of the connectivity distribution of the natural and the emerging networks indicates that both connectivity distributions are not significantly different (chi square test: *p* ≍ 0.085; see Materials and Methods). This result indicates that although the connectivity of the networks emerging in our simulation is not consistent with that of large metabolic networks described in the literature, it is similar to the connectivity observed in natural metabolic subnetworks of the same size. Note, however, that these subnetworks typically do not produce all compounds required for biomass formation and therefore have been selected for functions different from the networks emerging in our simulations.

**Figure 3:**
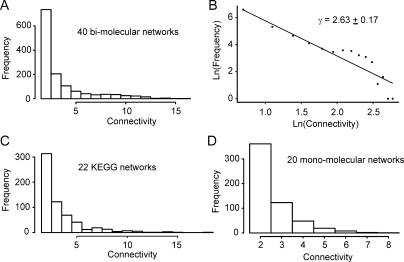
Connectivity Distribution of Simulated and Natural Networks (A) Connectivity distribution of 40 different simulated networks. As in the sample simulation, most metabolites are only involved in two or three reactions. On the other hand, the distribution shows that there are a number of hubs that are involved in up to 16 reactions. (B) The double-logarithmic plot of the distribution reveals a considerable deviation from a power-law distribution. (C) Connectivity distribution in natural metabolic networks of similar size. The networks are generated from the KEGG database for *E. coli* (see Materials and Methods). The connectivity distribution of the simulated networks is consistent with that of the natural networks (chi square test: *p* ≍ 0.085; see Materials and Methods). (D) Simulations for monomolecular reaction networks. The initial network is equivalent to the group transfer networks (see Figure 1C). In contrast to the group transfer network, we assume that groups are not transferred, but are added or removed by the enzymes. Therefore a reaction can transform a donor into its corresponding acceptor without being coupled to another half-reaction. The connectivity distribution of the emerging networks clearly differs from the connectivity distribution in the bimolecular group transfer networks. In particular, hubs are comparatively rare. This indicates that group transfer plays a key role in the emergence of hubs.

To analyze whether group transfer plays a major role in the emergence of hubs, we performed additional simulations with monomolecular reaction networks. In contrast to the bimolecular group transfer networks described above, we assumed that groups are not transferred between metabolites, but are simply added or removed by enzymes. The topology of the resulting initial network is similar to the group transfer network (see Figure 1C and 1D). However, in contrast to group transfer networks, in monomolecular networks there are no half-reactions. For simulating the evolution of monomolecular networks, we used assumptions analogous to those used for the simulations of the bimolecular group transfer networks. An example for an emerging monomolecular network is shown in Figure 2D. Note that this network produces the same set of metabolites involved in biomass formation as the network shown in Figure 2C. However, the resulting monomolecular reaction network differs remarkably from the bimolecular reaction network. In contrast to the group transfer reactions, monomolecular reactions have only a single substrate and product. Therefore monomolecular networks can be displayed as graphs that typically have a tree-like structure (see Figure 2D). The connectivity distributions of the simulated monomolecular networks clearly differs from the simulated group transfer networks (Figure 3D). In particular, monomolecular networks have much fewer hubs.

Our simulation results indicate that group transfer plays a key role in the emergence of hubs. Within the evolution of group transfer networks, hubs emerge because some metabolites become a preferential donor or acceptor of a specific group, i.e., they have a particularly high group transfer potential. Therefore it is of advantage that an enzyme that transfers this specific group increases its specificity towards this donor (or acceptor). Once a preferential donor (or acceptor) emerges and the corresponding enzyme starts to specialize towards this metabolite, the remaining enzymes in the network are selected for further increasing the group transfer potential of this specific metabolite. Thereby a single metabolite may “monopolize” this group transfer reaction. It serves as the only donor (or acceptor) of a specific group towards a number of different acceptors (donors) in the network. As a consequence it participates in a number of different reactions. In the absence of group transfer reactions, i.e., in monomolecular reaction networks, this mechanism does not work. Therefore, hubs do not emerge in monomolecular reaction networks.

## Discussion

In summary, our simulations demonstrate that metabolic networks may have emerged according to the scenario of Kacser and Beeby [5]. Starting from metabolic networks with multifunctional enzymes, networks emerge that consist of highly specialized enzymes. The key evolutionary mechanism in this scenario is the duplication of enzymes followed by the specialization of the copies for different biochemical reactions. The implication of this mechanism is that during the early evolution of metabolic networks, biochemical reactions and intermediate metabolites have been lost.

On the basis of our model, we expect that multifunctional enzymes can be maintained if they have only little impact in biomass formation and are therefore present as single copies. Alternatively, multifunctional enzymes can be maintained if there is no trade-off between the different functions. Recent results on the evolution of multifunctional enzymes indicate that this may at least be sometimes the case [13].

Although the overall connectivity of the networks emerging in our simulations differs clearly from a power-law distribution, it is consistent with the connectivity distribution derived from natural subnetworks of similar size. Our simulations indicate that the scenario of Kacser and Beeby leads to the emergence of hub metabolites. Additionally, our results predict that group transfer is essential for the emergence of hubs in these networks. This prediction is in line with the observation that most hubs in natural networks such as ATP, NADH, glutamate, and coenzyme A are key compounds in the transfer of biochemical groups. The high connectivity of these metabolites results mainly from transferase reactions rather than other reaction types (such as isomerase reactions).

In our simulations, hubs emerge as a consequence of selection for growth rate. In addition, along with a number of further publications given below, our simulations therefore support the view that hubs are not necessarily a result of selection for robustness as has been suggested previously [1,14]. First, it has been shown that the evolution of robustness against complete knock-outs of genes, as for example by duplicates, requires very specific conditions in terms of mutation rates, gene functions, and interactions [15]. Second, a recent study on robustness and enzyme indispensability in yeast metabolism indicates that the apparent dispensability of many enzymes is not due to network robustness, but to the fact that many enzymes are only required under specific environmental conditions [16]. Third, robustness against environmental changes is also unlikely to explain the connectivity distributions observed in natural networks. This is because power-law connectivity distributions have been observed in a wide range of organisms living in very different environments, including, for example, intercellular parasites that live in very stable environments [3]. Finally, no evolutionary scenarios have been presented that demonstrate that selection for increased robustness leads to the emergence of metabolic networks with power-law connectivity. Nevertheless, it has been shown that robustness of metabolic networks against partially deleterious mutations (such as a 50% reduction of gene dosage due to a defective allele in diploid organisms) may emerge as a “side product” of selection for growth rate [17,18]. It can therefore be expected that the networks emerging in our simulations are robust against slightly deleterious mutations.

## Materials and Methods

### Metabolites

Metabolites are assumed to consist of seven different biochemical groups. Each biochemical group is either present once or is absent. Thus there are 2^7^ different metabolites that are characterized by a binary string of length 7, where “1” at position *x* denotes the presence of group *x*, whereas “0” denotes the absence of the group. Metabolites are associated with a random free energy that is taken from a uniform distribution between zero and one, and is required to specify thermodynamic properties of the biochemical reactions. For the production of biomass, it is necessary to have at least one donor and acceptor of each group as external metabolites. Thus a minimal set of two metabolites, namely X0 (characterized by the string 0–0–0–0–0–0–0) and X127 (characterized by the string 1–1–1–1–1–1–1), is assumed to be present in the environment. Sixteen randomly chosen metabolites are involved in biomass formation.

### Enzymes

Enzymes catalyze the transfer of a specific biochemical group. We assume that groups are transferred by a “ping-pong mechanism” (see Figure 1): A donor of a group transfers the group to the appropriate enzyme and is thereby transformed into its corresponding acceptor. The enzyme then transfers the group to an acceptor, thereby transforming it into its corresponding donor. Thus an enzyme can be in two possible states, *E_i_* ^(1)^ and *E_i_* ^(0)^, with *E_i_* ^(1)^ *+ E_i_* ^(0)^ *= E_i_ ,* where *E_i_* is the total dosage of enzyme *i,* *E_i_* ^(0)^ is the concentration of the enzyme without its group being bound to it, and *E_i_* ^(1)^ is the concentration of enzyme with its group being bound to it. The free energy difference between both states of an enzyme is assumed to be a random value taken from a uniform distribution ranging from zero to one. We further assume that in principle all metabolites that contain a specific biochemical group (i.e., half of the 2^7^ metabolites) can serve as a donor for group transfer, whereas all metabolites that do not contain the group can in principle serve as an acceptor. We assume linear kinetics for the transfer of a group to an enzyme, given by *v = k_ij _*(*E_i_* ^(0)^ *X*_(*ij*)_^(1)^ *−E_i_* ^(1)^ *X*_(*ij*)_^(0)^ */q_ij_*), where *k_ij_* is the rate constant of the reaction *j* of an enzyme *i,* *X*_(*ij*)_^(1)^ is the concentration of the donor of the reaction, *X*_(*ij*)_^(0)^ is the concentration of the corresponding acceptor, and *q_ij_* is the equilibrium constant of the reaction resulting from the free energies of the reactants. For the transfer of a group from a donor to an acceptor, two half-reactions need to be coupled. This results in Michaelis-Menten–like kinetics and implies that functional enzymes need to maintain nonzero rate constants for at least two reactions. We assume that initially the enzymes are unspecific and transform groups from each donor to each acceptor with the same rate constant. The initial dosage of enzymes is *E_i_ =* 1.

### Transporters

We further assume that transporters transport metabolites passively across the cell membrane. The rate of transport is given by *v = T_i_t_ij_*(*X_j_−X_j_^ext^*)*,* where *T_i_* is the dosage of the transporter *i,* *t_ij_* is the rate constant of the transport of metabolite *j,* *X_j_* is the metabolite concentration in the cell, and *X_j_^ext^* is the metabolite concentration in the environment. We assume that in the initial network there is a single transporter that transports all metabolites with the same rate constant. The initial dosage of the transporter is *T*_0_ *=* 1.

### Biomass formation, growth, and network fitness

Biomass is formed by the condensation of specific metabolites. The rate of biomass formation follows linear kinetics given by the product *v_BM_ = k_BM_Π_i_X_i_* over all metabolites *X_i_* that are involved in biomass formation. The rate constant is set to *k_BM_ =* 1 in all simulations. We assume that the formation of biomass leads to growth. The growth rate is given by *W =* 1*/VdV/dt = v_BM_ /*(*C*_0_ *+C_E_E+C_T_T*), where *C*_0_ is the amount of biomass that is required for structural compounds (i.e., those compounds that are not directly involved in cellular metabolism), *C_E_* is the amount of biomass per enzyme, *C_T_* is the amount of biomass per transporter, *E* is the total dosage of enzymes, and *T* is the total dosage of transporters [5]. The parameters *C*_0_, *C_E_*, and *C_T_* are set to 50, 1, and 1, respectively. Note that due to cell growth, metabolites are constantly diluted at a rate equal to the growth rate. The fitness of a network is assumed to be proportional to the steady-state growth rate.

### Tradeoff between specificity and catalytic activity

In line with the scenario of Kascer and Beeby, we assume that reactions can either catalyze a large number of reactions but with low activity for each enzyme, or a lower number or reactions with improved catalytic activities. Specifically, we assume that the sum Σ*_j_ k_ij_* ^1*/α*^ and Σ*_j_ t_ij_* ^1*/α*^ over all rate constants *k_ij_* or *t_ij_* of an enzyme or transporter, *i*, respectively, is constant. For values of *α >* 1 increasing the rate constant for a single reaction has an over-proportional effect on all other rate constants. In our simulations we use Σ*_j_ k_ij_* ^1*/α*^ *=* 1, Σ*_j_ t_ij_* ^1*/α*^ *=* 1, and *α* *=* 2. This implies, for example, that a transporter catalyzing the transport of a single metabolite has a four times higher rate constant for this reaction than a transporter that is specialized on the transport of two metabolites.

### Mutations and the course of evolution

We assume that there are two types of mutations: (1) mutations that change the kinetic properties of an enzyme and (2) mutations that change the number of copies of an enzyme, i.e., gene deletions and duplications. For the first type of mutation we assume that the value of *k_ij_* ^1*/α*^ or *t_ij_* ^1*/α*^ *,* respectively, for a single reaction is either increased or decreased by a small value of *m =* 0*.*01, while the rate constants of the other reactions are decreased, or increased appropriately. Gene deletions decrease the dosage of an enzyme. Duplications increase the dosage of an enzyme. In our simulations we calculate the effect of all possible mutations of the current network on the steady-state growth rate to obtain the mutant with maximal increase in fitness. This mutation is then assumed to become fixed and the resulting network is used to search for the next mutations. The steady states are calculated using the NAG library function *c05nbc* (see http://www.nag.co.uk/numeric/CL/CLdocumentation.asp for documentation) for root finding of systems of nonlinear equations. The solver may fail (or may obtain steady states with negative concentrations) if the initial approximation of the steady state is far away from the actual steady state of the system. In this case we integrated the system towards its steady state (using the NAG library function *d02ejc*) until the root finder succeeds. To calculate the effect of a mutation on the steady state, we solve the mutated system using the metabolite concentrations of the non-mutated system as initial approximation, because for small mutations, the steady state of the mutated systems can be expected to be close to the non-mutated system. Note, however, that in a system of nonlinear equations there may be multiple steady states, unstable steady states, and saddle points. We therefore test whether the steady state of the most beneficial mutation obtained by the above procedure (i.e., the mutation that is assumed to become fixed) is reached by integration from initial metabolite concentrations close to zero. This test failed in ten out of 50 simulations. These simulations are excluded from further analysis. Gene duplications and deletions are assumed to be rare compared to mutations affecting the catalytic properties of enzymes and transporters. We therefore consider these types of mutation only if none of the mutations affecting kinetic properties are beneficial. A simulation ends if there are no beneficial kinetic mutations nor gene duplications or deletions.

### Analysis of *E. coli* metabolic subnetworks

We analyzed the connectivity in metabolic subnetworks of *E. coli* downloaded from the KEGG database (ftp.genome.ad.jp/pub/kegg/ pathways/eco/) on the basis of the annotation in the reaction database (ftp.genome.ad.jp/ pub/kegg/ligand/reaction). Metabolites participating in fewer than two reactions are excluded. For comparison with the networks emerging in our simulation, we included 22 subnetworks of similar size, i.e., with between 24 and 40 metabolites. To compare the connectivity distributions, we use Pearson's chi square test. Data from the tail of the distributions (*k >* 11) are put into a single class.

### Simulation of monomolecular networks

For simulating the evolution of monomolecular networks, we assume that enzymes add or remove biochemical groups, thereby transforming a donor of a group into its corresponding acceptor and vice versa. In contrast to bimolecular reactions, it is not required to couple two half-reactions. Completely specialized enzymes catalyze a single reaction rather that two half-reactions. The rate of a reaction is given by *v = E_i_ k_ij_*(*X*_(*ij*)_^(1)^ *−X*_(*ij*)_^(0)^ */q_ij_*), where *k_ij_* is the rate constant of the reaction *j* of an enzyme *i*, *E_i_* is the dosage of the enzyme, *X*_(*ij*)_^(1)^ is the concentration of the donor, *X*_(*ij*)_^(0)^ is the concentration of the corresponding acceptor, and *q_ij_* is the equilibrium constant of the reaction resulting from the free energies of the reactants. All other assumptions are equal to those used in the simulations of the bimolecular group transfer networks.
